# Application of Queuing Analytic Theory to Decrease Waiting Times in Emergency Department: Does it Make Sense?

**DOI:** 10.5812/atr.11376

**Published:** 2013-12-01

**Authors:** Sean Shao Wei Lam, Marcus Eng Hock Ong

**Affiliations:** 1 Health Services Research Unit, Singapore General Hospital, Singapore; 2 Department of Emergency Medicine, Singapore General Hospital, Singapore

**Keywords:** Emergency Department, Operations Research, Simulation, Quality Improvement

Dear Editor,

We read with great interest the recent manuscript by Mostafa Alavi-Moghaddam et al. (1) on “Application of Queuing Analytic Theory to Decrease Waiting Times in Emergency Department: Does it Make Sense?” published in the Archives of Trauma Research. In this article, the authors described a discrete events simulation (DES) model deployed on the ARENA simulation software to model the processes in an Emergency Department (ED). The simulation model was based on empirical data of actual ED operations for The Imam Hosein Hospital located in Tehran, Iran, with annual input of approximately 50,000 patients, 24 general emergency beds and 14 trauma emergency beds.

The application of simulation models for improving EDs’ work processes started in the late 1980s (2) and have been gaining traction over the last decade (3-11). The objectives of simulation modeling for EDs have been varied, ranging from infrastructural designs (3), process improvements (4-6, 12), staffing decisions (10) and near-term forecasting for proactive operational management (11). Simulation models in general can be developed for different hospital processes under a variety of settings. However, differences in operating parameters, such as process designs and infrastructural configurations, prevent easy generalizability of simulation models, associated results and policy insights. Different objectives may also entail different modeling assumptions and data requirements. It should be noted that the ability for any simulation model to faithfully represent the actual system have to be justified through rigorous model validation and verification processes before they can be used for scenario analysis and policy testing (13).

The authors described a set of scenarios related to manpower resource management (e.g. nurse scheduling) and capacity improvements (e.g. changes to diagnostic, laboratory, consultation and discharge capacities) using the simulation model. Forecasting accuracy is critical in such simulation models, since the quality of realized performance hinges upon the accuracy of forecasts. To a certain extent, scenario analytic techniques may be able to circumvent this issue by presenting a plethora of plausible scenarios with sufficiently wide coverage thereby facilitating the development of policies robust against the identified scenarios. Estimates of individual parameters describing the ED system are integrated within DES models that capture the interlocking internal dynamics within complex ED systems (11, 14). As demonstrated in the study, the internal dynamics of ED systems can be captured through the use of DES models to provide a viable decision support tool.

The simulation model examined typical input, throughput and output factors influencing ED overcrowding and reported encouraging results. The mean waiting times at the various processes and average length of stay (LOS) within the ED have been reported as the primary outcome measures. Other outcome measures, such as utilization levels for beds or care units (11), probability of ambulance diversion (11, 15, 16), ED productivity (17) and the proportion of patients who leave without being seen (LWBS) (18), can also be of interest to decision makers. 

Empirical data is critical for the development of a robust simulation model that considers fluctuating demands and dynamic resource allocation decisions which characterizes ED operations. Characteristics of patient demands (e.g. triage categories, reneging characteristics and medical/ trauma categories) and the historical distributions of pertinent time intervals (e.g. patient inter-arrival times and service times of the various ED processes) are important parameters that have to be carefully modeled. Accurate and updated spatiotemporal patient flow data may not be readily captured via the data-collection scheme described in the paper. Real-time location systems (RTLS) for tracking the status and movement of various entities within the ED (e.g. patients, healthcare workers and physical assets) (19) may improve the quality of data, and hence, the accuracy of forecasts.

Simulation modeling provides a rigorous and versatile platform for evidence-based decision making in ED process improvements, infrastructural designs and policy evaluations. Simulation models have been shown to provide for short term operational, tactical and strategic ED planning and management. Different study objectives necessitate different modeling assumptions, data resolution and data quality requirements. As ED overcrowding has been identified as a significant international crisis that is detrimental to patient safety, quality of care and patient satisfaction (20), there is an immediate need to re-examine and improve ED processes using rigorous evidence-based and quantitative modeling techniques. In this respect, it is encouraging to see the increasing adoption of simulation to quantitatively model the inherent complex system dynamics present in EDs around the world.
